# Foxj2 Attenuates LPS‐Induced Inflammatory Response in Macrophages

**DOI:** 10.1155/mi/3854538

**Published:** 2025-12-11

**Authors:** Pianpian Huang, Jun Fu, Ji Hu, Yinghong Lei, Caobo Dai, Tingyu Wu, Ju Liu

**Affiliations:** ^1^ Department of Geriatrics, Wuhan No.1 Hospital, Wuhan, 430000, Hubei, China, whyyy.com; ^2^ Department of Radiology, Wuhan No.1 Hospital, Wuhan, 430000, Hubei, China, whyyy.com; ^3^ Department of Cardiology, Renmin Hospital of Wuhan University, Wuhan, 430000, Hubei, China, rmhospital.com

**Keywords:** forkhead box J2, inflammation, inflammation signaling pathway, LPS, macrophage

## Abstract

**Background:**

Macrophages are central to innate immune responses and are crucial in maintaining homeostasis and managing inflammatory diseases. Forkhead box J2 (Foxj2) is a member of the forkhead/hepatocyte nuclear factor 3 transcription factor family and is essential for multiple biological functions. However, the involvement of Foxj2 in the inflammatory process in macrophages remains unclear.

**Objective:**

The present study aimed to explore the role of Foxj2 in the inflammatory processes of macrophages activated through lipopolysaccharide (LPS) stimulation.

**Methods:**

The modulation of Foxj2 expression in macrophages in response to LPS stimulation was investigated via reverse‐transcription quantitative (RT‐q) PCR, Western blot, and immunofluorescence staining assays. Macrophages were infected with adenovirus vectors to upregulate the expression of the Foxj2 gene. Luciferase reporter gene assay and chromatin immunoprecipitation (ChIP)‐PCR analysis were used to determine the regulatory relationship between Foxj2 and Tak1 (transforming growth factor‐β‐activated kinase 1).

**Results:**

LPS stimulation of peritoneal macrophages led to a significant decrease in Foxj2 expression. In addition, LPS treatment led to Foxj2 depletion in several mouse tissues, including the heart, liver, spleen, lungs, kidneys, adipose tissue, blood vessels, and peritoneal macrophages. Furthermore, Foxj2 overexpression ameliorated the mRNA expression of TNF, IL‐1β, IL‐6, IL‐12, IFN‐stimulated gene 15, and IFN‐β in macrophages treated with LPS. Additionally, Foxj2 overexpression attenuated phosphorylation of Stat1, p65, Erk1/2, Jnk, and p38. Subsequent experiments confirmed the binding of Foxj2 to the promoter region of Tak1, led to the suppression of Tak1’s transcriptional activity. Moreover, a reduction in Foxj2 levels was observed during the pathological processes of numerous diseases characterized by inflammation, including high‐fat diet (HFD)–induced obesity, HFD‐induced nonalcoholic fatty liver disease (NAFLD), doxorubicin‐induced cardiomyopathy, acute myocardial infarction (AMI) and D‐galactose induced aging conditions.

**Conclusion:**

The present findings indicated that Foxj2 is crucial in mitigating macrophage inflammation induced by LPS and might be considered a target for treating sepsis and other inflammatory diseases.

## 1. Introduction

The innate immune system is uniquely positioned to counteract the invasion of infectious microbes. However, it can also initiate pathological mechanisms that underpin a range of diseases such as sepsis, atherosclerosis, chronic inflammation, obesity, autoimmune disorders, cancer, and the aging process [1–3]. Macrophages are instrumental in orchestrating the immune response, with their activation being a central element in the emergence of several diseases [4]. The factors that are present in the tissue microenvironment are instrumental in directing the macrophages’ polarization into the two main phenotypes, namely the M1 type, which is classically activated, and the M2 type, which is alternatively activated [5]. M1 macrophages, through the production of proinflammatory mediators, can lead to rampant inflammation and subsequent organ failure [6]. Conversely, M2 macrophages contribute to the process of tissue repair and remodeling by secreting anti‐inflammatory factors [6]. Therefore, the balance of macrophage polarization plays a pivotal role in the pathophysiology of diseases with an inflammatory component and its regulation represents a potential therapeutic strategy for their management.

The forkhead/hepatocyte nuclear factor 3 family of transcription factors, characterized by a conserved DNA‐binding domain known as the “forkhead” or “winged‐helix”, is extensive and is involved in various biological processes [7–9]. These factors exert their functions by modulating the expression of diverse target genes [10, 11]. In the murine central nervous system of mice, forkhead transcription factors are implicated in cell differentiation, developmental processes, and lifespan regulation [12–16]. Moreover, their pivotal role in the modulation of immune responses is well‐documented [17].

Forkhead box J2 (Foxj2), a less‐explored member of the forkhead family, exhibits dual sequence specificity in DNA binding [11]. Foxj2 can be expressed as two differently sized isoforms, with differences in their C‐termini. These isoforms are likely encoded by a single gene on chromosome 12 through alternative splicing. The larger isoform has an extra acidic transactivation domain at its C‐terminus, which might explain the functional differences. The two isoforms seem to be expressed differently in various cell lines [18]. A previous study indicated that the expression of Foxj2 is elevated during spermatogenesis and the earliest stages of embryonic development [19]. Foxj2 is involved in cell proliferation, differentiation, migration, and stem cell maintenance [20]. In recent years, some studies started to uncover the link between Foxj2 and inflammation. For example, Foxj2 could potentially contribute to the pathophysiological process of spinal cord injury by regulating the production of inflammatory mediators in astrocytes [21]. Foxj2 accelerates the pathogenesis of antiphospholipid syndrome by inducing inflammation [22]. However, the potential contributions of Foxj2 to inflammation remain unclear.

By investigating the expression and distribution patterns of Foxj2 in a lipopolysaccharide (LPS)–induced mouse model of sepsis, the present authors aimed to enhance the current understanding of the physiological functions of Foxj2 in the context of inflammation and to explore its link to macrophage polarization and the inflammatory signaling cascade. The present findings could provide valuable insights into the complex interplay between immune response modulation and disease progression, potentially facilitating the discovery of innovative therapeutic targets for treating diseases associated with inflammatory conditions.

## 2. Materials and Methods

### 2.1. Mice

Male C57BL/6 mice, aged 8–12 weeks, were bred and housed in standard conditionsin the Tongji Medical School Experimental Animal Center. The endotoxemia model was established in mice by administering an intraperitoneal dose of 25 mg/kg LPS (*Escherichia coli* 0111: B4; Sigma‐Aldrich; Merck KGaA). Control mice were also injected with an equal volume of sterile normal saline. At various time points following LPS injection, mice were euthanized through cervical dislocation following anesthesia with intraperitoneal injection of sodium pentobarbital (50 mg/kg). The levels of inflammatory factors in mice were measured 6 and 24 h after LPS injection, respectively. Finally, at 24 h postinjection, the mice were euthanized to proceed with the histological evaluation of tissue samples. The in vivo experiments were conducted with the approval of the Ethics Committee of Tongji Medical College, Huazhong University of Science and Technology (IACUC Number 4012) and adhered to National Institutes of Health guidelines for animal care and use.

### 2.2. Cell Culture

The isolation of peritoneal macrophages from C57BL/6 mice was performed following a standard protocol [23]. The process began with the lavage of the peritoneal cavity using 6 mL sterile sodium chloride (NaCl) solution at a concentration of 0.9%. This solution was used to flush the cavity and the resulting lavage fluids were collected, combined, and centrifuged to separate the cells from the fluid. Cell pellets obtained after centrifugation were resuspended in RPMI1640 medium (Gibco; Thermo Fisher Scientific, Inc.), enriched with 10% FBS (Gibco; Thermo Fisher Scientific, Inc.). Macrophages were then seeded into culture plates and allowed to adhere to the plate surface for 2 h. After this incubation period, any nonadherent cells, which typically include nonmacrophage cells, were removed by washing the plates with a sterile solution. The adherent macrophages, comprising a relatively pure population, were cultured for an additional 24 h in RPMI1640 medium supplemented with 10% FBS for further study. In parallel, RAW264.7 cells, a well‐characterized murine macrophage cell line, were obtained from the American Type Culture Collection (ATCC). These cells were cultured in complete DMEM containing 10% FBS for further study.

### 2.3. Experimental Design

To study the effect of LPS on the mRNA expression level of Foxj2, peritoneal macrophages were stimulated with 1 μg/mL LPS for 0, 1, 2, 6, 12, and 24 h and the mRNA and protein levels of Foxj2 were measured in each group. To find the appropriate stimulation concentration, peritoneal macrophages were stimulated with LPS at 0, 1, 5, 10, 50, and 100 μg/mL, respectively for 24 h and the protein level of Foxj2 was measured in each group. Peritoneal macrophages were stimulated with 1 μg/mL LPS for 24 h and the expression and nuclear translocation of Foxj2 were detected through immunofluorescence. To examine the effect of Foxj2 on the LPS‐induced endotoxemia model, peritoneal macrophages were infected with adenovirus‐empty vector (Ad‐EV) or adenovirus‐Foxj2 (Ad‐Foxj2) 48 h after being treated with or without LPS (1 μg/mL). The expression of inflammatory markers was measured after treatment with LPS for 4 or 20 h. The secretion of inflammatory markers was measured after treatment with LPS for 24 h by enzyme‐linked immunosorbent assay (ELISA). To examine the effect of Foxj2 on LPS‐induced inflammatory signaling pathways, the macrophages were treated with LPS for 0.5, 2, 6, and 10 h, respectively, and the level of signaling pathway phosphorylation was measured. To investigate the impact of IL4 on Foxj2 protein expression levels, peritoneal macrophages were treated for 48 h with IL4 (Catlog Number HY‐P7080; MedChemExpress) at concentrations of 0, 1, 5, 10, 20, and 50 ng/mL and Foxj2 protein levels were assessed in each group. To determine the temporal dynamics of IL4‐induced Foxj2 protein expression, peritoneal macrophages were stimulated with 20 ng/mL IL4 for 0, 2, 6, 12, 24 and 48 h, and the Foxj2 protein levels were measured at each time point. Furthermore, to assess Foxj2’s role in IL4‐mediated M2 polarization of macrophages, peritoneal macrophages were infected with either Ad‐EV or Ad‐Foxj2 for 48 h, followed by treatment with or without 20 ng/mL IL4. The mRNA expression of M2 markers and cytokines was then evaluated at 4 and 20 h after IL4 treatment.

### 2.4. RNA Extraction and Reverse‐Transcription Quantitative (RT‐q) PCR

Cellular or tissue‐derived total RNA was extracted with the TRIzol reagent (Invitrogen; Thermo Fisher Scientific, Inc.). For cDNA synthesis, the PrimeScript RT Master Mix kit (Takara Bio, Inc.) was employed and the subsequent qPCR was performed with SYBR Green Master Mixture (Takara Bio, Inc.) on the ABI StepOnePlus RT‐PCR system (Applied Biosystems; Thermo Fisher Scientific, Inc.). To quantify relative gene expression, including Foxj2 and several other host inflammatory genes, normalization against the internal control gene β‐actin was performed using the comparative Cq method as expressed by the formula 2^−ΔΔCq^. The sequences of primers utilized for the qPCR assays were as follows:

5′‐CGTTGACATCCGTAAAGACC‐3′ and 5′‐AACAGTCCGCCTAGAAGCA‐3′ for β‐actin;
5′‐GCCTCCGACCTGGAGAGTAG‐3′ and 5′‐CTGTACCGTGGCTTGCCAT‐3′ for Foxj2;
5′‐GCAACTGTTCCTGAACTCAACT‐3′ and 5′‐ATCTTTTGGGGTCCGTCAACT‐3′ for IL‐1β;
5′‐TAGTCCTTCCTACCCCAATTTCC‐3′ and 5′‐TTGGTCCTTAGCCACTCCTTC‐3′ for IL‐6;
5′‐CTGTGCCTTGGTAGCATCTATG‐3′ and 5′‐GCAGAGTCTCGCCATTATGATTC‐3′ for IL‐12;
5′‐CCCTCACACTCAGATCATCTTCT‐3′ and 5′‐GCTACGACGTGGGCTACAG‐3′ for TNFα;
5′‐GGTGTCCGTGACTAACTCCAT‐3′ and 5’‐TGGAAAGGGTAAGACCGTCCT‐3′ for ISG15;
5′‐AGCTCCAAGAAAGGACGAACA‐3′ and 5′‐GCCCTGTAGGTGAGGTTGAT‐3′ for IFNβ.
5′‐CTCCAAGCCAAAGTCCTTAGAG‐3′ and 5′‐GGAGCTGTCATTAGGGACATCA‐3′ for Arg1;
5′‐CTCTGTTCAGCTATTGGACGC‐3′ and 5′‐CGGAATTTCTGGGATTCAGCTTC‐3′ for CD206;
5′‐CAATGTGGTTAGTTGGATCGGC‐3′ and 5′‐CCCAGTTCTTAAAGCCTTTCTCA‐3′ for CD301;
5′‐CTCTGCCATCACGTTTAGTGAA‐3′ and 5′‐GACGGTTATCAAAACAACGCC‐3′ for CCL22;
5′‐ATTCTGTGACCATCCCCTCAT‐3′ and 5′‐TGTATGTGCCTCTGAACCCAC‐3′ for CCL24;
5′‐TTCTTCGATTTGGGTCTCCTTG‐3′ and 5′‐GTGCAGCTCTTGTCGGTGAA‐3′ for CCL26.

### 2.5. Western Blotting

Total cellular proteins were extracted using the RIPA lysis buffer at 4°C for 30 min, followed by their quantification utilizing a BCA kit. A total of 60 µg of protein was separated by 10% SDS‐PAGE and transferred onto a PVDF membrane, which was then blocked for 2 h at ambient temperature using a blocking solution made of 5% milk in a TBS‐Tween buffer with a concentration of 0.1%. The membranes were subjected to overnight incubation at 4°C with with a series of primary antibodies including: anti‐β‐actin (diluted 1:1000; Catalog Number 66009–1–Ig, sourced from Proteintech, Wuhan, China); anti‐Foxj2 (1:1000 dilution; product code sc‐514265, supplied by Santa Cruz, USA); anti‐Stat1 (1:200 dilution; product code sc‐51702, Santa Cruz, USA); antiphospho‐Stat1 (1:200 dilution; Catalog Number sc‐136229, Santa Cruz, USA); anti‐NF‐κB p65 (1:1000 dilution; Catalog Number 8242, Cell Signaling Technology, MA, USA); anti‐phospho‐NF‐κB p65 (Ser468) (1:1000 dilution; Catalog Number 3039, CST); anti‐Jnk (1:200 dilution; sc‐81502, Santa Cruz, USA); anti‐phospho‐Jnk (1:200 dilution; sc‐62547, Santa Cruz, USA); anti‐Erk (1:200 dilution; sc‐65981, Santa Cruz, USA); anti‐phospho‐Erk (1:200 dilution; sc‐7383, Santa Cruz, USA); anti‐p38 (1:1000 dilution; product code A10832, Abclonal); and finally, anti‐phospho‐p38 (1:1000 dilution; Catalog Number AP0056, Abclonal). Subsequently, the membranes underwent a 2h incubation at room temperature with the appropriate horseradish peroxidase‐conjugated secondary antibodies. The subsequent visualization of the protein bands was achieved using a chemiluminescence detection system.

### 2.6. Histology Analysis

Mouse lung and adipose tissues were subjected to fixation overnight using a 4% solution of paraformaldehyde and subsequently embedded in paraffin wax. Thereafter, tissue specimens were cut into 5 µm sections and dewaxed with xylene, followed by a series of rehydration steps with decreasing ethanol concentrations. For histological evaluation, the sections were stained with the traditional H&E procedure.

### 2.7. Immunofluorescence

Immunofluorescent staining was performed following previously established protocols. The paraffin‐embedded slides were first dewaxed and thereafter incubated with primary antibodies directed against Foxj2 (1:50; Catlog Number ab54210; Abcam) and CD68 (1:50; Catlog Number ab109199; Abcam) overnight at 4°C. After primary antibodies incubation, the slides underwent a thorough wash cycle, after which they were exposed to fluorescein and tetramethyl rhodamine–conjugated secondary antibodies for 1 h at ambient room temperature. Upon completion of the secondary antibody binding, the slides were stained with DAPI for nuclear visualization. Subsequently, the mounted slides were observed using an epifluorescence microscope, capturing three randomly chosen fields for each sample. The immunofluorescent signals were meticulously documented using an epifluorescence microscope.

### 2.8. ELISA

Cells in 100 μL of medium were seeded onto 96‐well plates and treated under different conditions. At the appropriate time, 100 μL of supernatants was harvested for ELISA assay according to the instruction of the manufacturer [24]. The ELISA kit used in this study was purchased from Jiangsu Meimian Industrial Co., Ltd.

### 2.9. Luciferase Reporter Assay

A Foxj2‐overexpressing vector and luciferase reporter gene vector containing the promoters for Tak1 were constructed. Luciferase reporter constructs were cotransfected with an internal control plasmid, pRL‐TK (Renilla luciferase reporter plasmid; Promega), into HEK293 cells [25], followed by infection with Ad‐Foxj2 or Ad‐EV. Then, cells were harvested, lysed, and the luciferase activity was determined with the Dual Luciferase Reporter Assay Kit (Promega), according to the manufacturer’s instruction.

### 2.10. Chromatin Immunoprecipitation (ChIP) Assay

ChIP was carried out by a ChIP assay kit (Millipore). Mouse IgG was used as the negative control. The fractured DNA samples were pulled down by a mouse monoclonal antibody against Foxj2 (sc‐514265). Subsequently, the enriched DNA fragments were detected by PCR using specific primers as follows:

5′‐CCATCTTGGGGTTCTCTGACC‐3′ and 5′‐GGGCACAGGCTTGAACCTTA‐3′ for Seq 1;

5′‐AATCTTGTTGGGTAGTCTTCAGGA‐3′ and 5′‐GACACACCAAGACGACGGAT‐3′ for Seq 2.

### 2.11. Statistical Analysis

Statistical analysis was conducted using GraphPad Prism software (GraphPad Software Inc.; Dotmatics). Data are presented as mean ± standard error of the mean (SEM) from a minimum of three experimental replicates. The Student’s *t*‐test was employed to determine statistical differences between two groups, while one‐way or two‐way ANOVA with Tukey’s post hoc test was used for multiple comparisons. *p*  < 0.05 was considered to indicate a statistically significant difference.

## 3. Results

### 3.1. Expression of Foxj2 in Macrophages Is Suppressed by LPS and Activated by IL4

To explore the potential involvement of Foxj2 in LPS‐induced inflammatory reactions within macrophages, the expression levels of Foxj2 in peritoneal macrophages upon LPS stimulation were examined using both RT‐qPCR and western blotting. The present findings revealed a substantial decline in the expression of Foxj2 following LPS stimulation. When macrophages were exposed to 1 μg/ml LPS, Foxj2 mRNA levels started to decrease noticeably after 2 h, reaching the lowest point after 6 h (Figure 1A). Through western blot analysis, due to the different expression levels, β‐actin and Foxj2 were exposed independently. It was demonstrated that a 24 h treatment with 1 μg/ml LPS effectively downregulated the Foxj2 protein expression (Figure 1B). Further investigation showed that at this same concentration, the Foxj2 protein level began to decline at 12 h, with a significant reduction at the 24 h time point (Figure 1C). Conversely, IL4 stimulation induced differentiation into alternatively activated macrophages, known as M2 macrophages, which display a distinct phenotype that promotes tolerance, and Th2 immune responses. Importantly, this IL4 stimulation led to a significant upregulation of Foxj2 protein expression (Supporting Information 1: Figure S1A,B). Immunofluorescence analysis indicated robust expression of Foxj2 in untreated peritoneal macrophages acting as controls, whereas a notably weaker expression was measured in macrophages that had been stimulated with LPS (Figure 1D,E). These collective data suggested a probable involvement of Foxj2 in mediating the inflammatory response triggered by LPS in macrophages.

Figure 1LPS stimulation inhibits Foxj2 expression in peritoneal macrophages. (A) The levels of Foxj2 expression in peritoneal macrophages were measured in vitro after treatment with LPS (1 μg/mL) at various time points (*n* = 5). (B) The protein expression level of Foxj2 in peritoneal macrophages exposed to various concentrations of LPS for 24 h. (C) Temporal expression pattern of Foxj2 protein in peritoneal macrophages cultured with LPS (1 μg/mL). (D) Immunofluorescent detection of Foxj2 (green) in LPS‐treated macrophages. DAPI was used for nucleus staining (blue). (E) Quantitative results of Foxj2 fluorescence intensity (*n* = 6). The data are presented as the mean ± SEM.  ^∗^ *p*  < 0.05 vs. control group. Foxj2, forkhead box J2; LPS, lipopolysaccharide.

**Figure:**
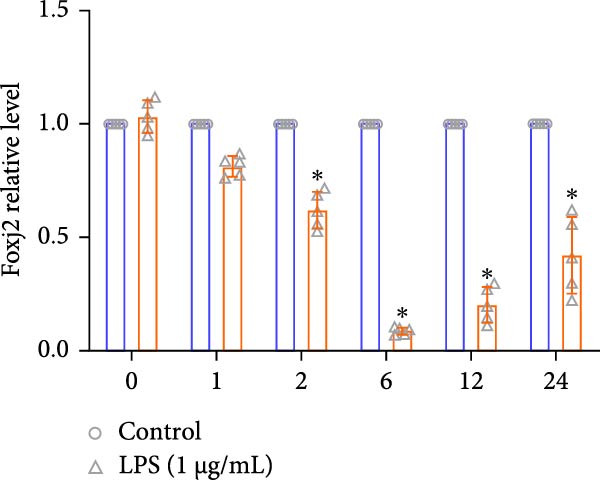
(A)

**Figure:**
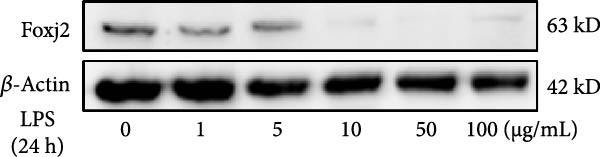
(B)

**Figure:**
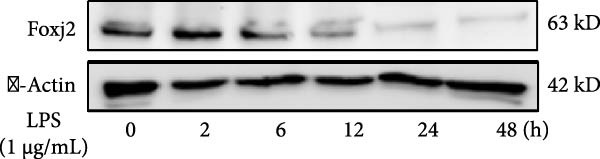
(C)

**Figure:**
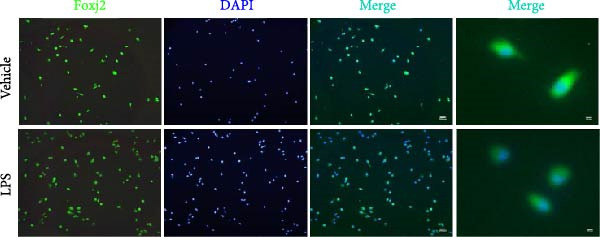
(D)

**Figure:**
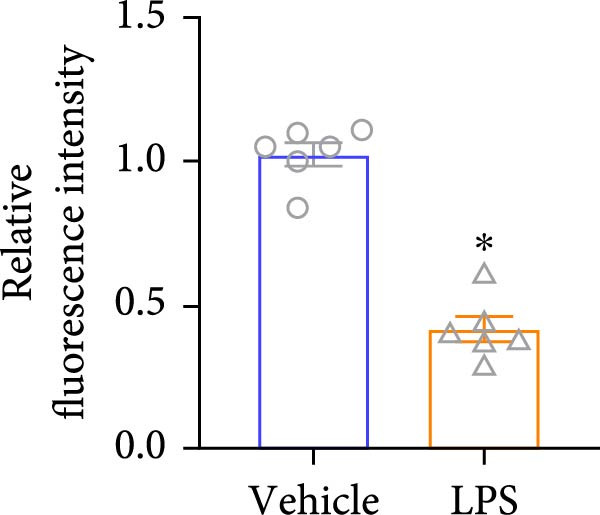
(E)

### 3.2. Foxj2 Expression Is Downregulated in Mice With Endotoxemia

An analysis of Foxj2 mRNA expression across different tissues in mice indicated that it is most prominently expressed in peritoneal macrophages, followed by the pulmonary, spleen, and heart tissues (Figure 2A). These findings are consistent with the outcomes of the current in vitro experiments, where it was found that administering LPS resulted in a time‐dependent reduction of Foxj2 mRNA levels across a variety of mouse tissues, such as the heart, liver, spleen, lungs, kidneys, adipose tissue, blood vessels, and peritoneal macrophages (Figure 2B). Further corroborating these observations, colocalization studies showed that similar to the pattern observed in adipose tissue (Figure 2C,D) and lung tissue (Figure 2E,F). In addition, the expression level of Foxj2 was significantly decreased in CD68‐positive macrophages isolated from the adipose tissue (Figure 2G,H) and lung tissue (Figure 2I,J) of mice treated with LPS. The present data indicate that the Foxj2 expression level is dynamically modulated depending on the polarization state of the macrophages.

Figure 2Foxj2 expression is downregulated in mice with endotoxemia. (A) Determination of Foxj2 mRNA expression patterns and tissue distribution in C57BL/6 mice. (B) The mRNA expression of Foxj2 was evaluated in various mouse tissues subjected to endotoxemia following a 25 mg/kg lipopolysaccharide induction at 6 and 24 h. (C) Immunofluorescence staining was employed to assess Foxj2 levels in adipose tissue of mice with and without endotoxemia. (D) Quantitative results of Foxj2 fluorescence intensity in adipose tissue. (E) Immunofluorescence staining was conducted to analyze the expression of Foxj2 in lung tissue from both normal and endotoxemic mice. (F) Quantitative results of Foxj2 fluorescence intensity in lung tissue. For colocalization studies, tissue sections were co‐stained with antibodies against Foxj2 (visualized in green) and CD68 (a macrophage marker; visualized in red). Nuclear staining was performed using DAPI (in blue). Arrows highlight cells that are double positive for Foxj2 and CD68. (G,H) The expression level of Foxj2 in CD68‐positive macrophages isolated from adipose tissue. (I,J) The expression level of Foxj2 in CD68‐positive cells isolated from lung tissue. Data are expressed as mean ± SEM (*n* = 6).  ^∗^ *p*  < 0.05 vs. control group. Foxj2, forkhead box J2.

**Figure:**
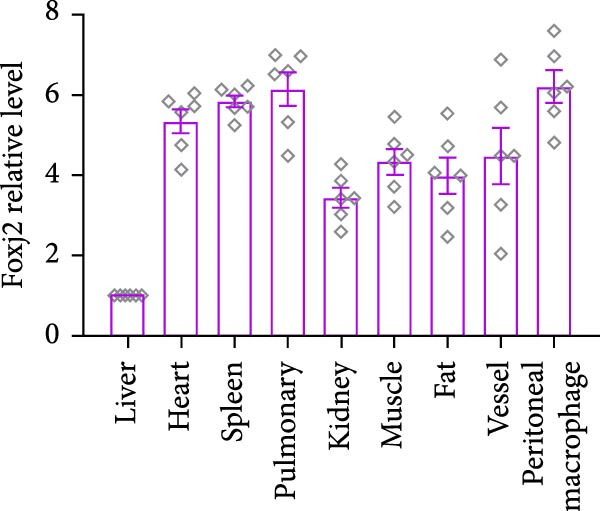
(A)

**Figure:**
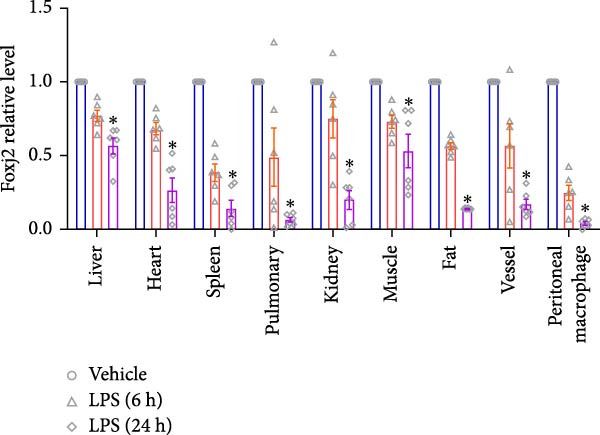
(B)

**Figure:**
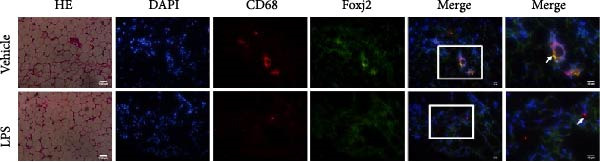
(C)

**Figure:**
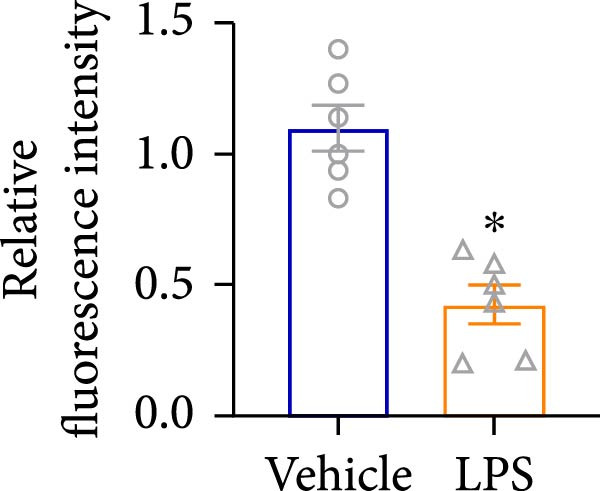
(D)

**Figure:**
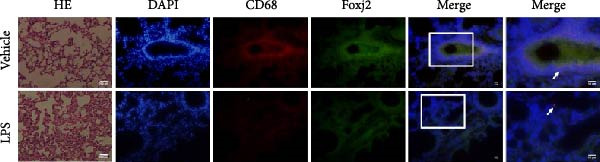
(E)

**Figure:**
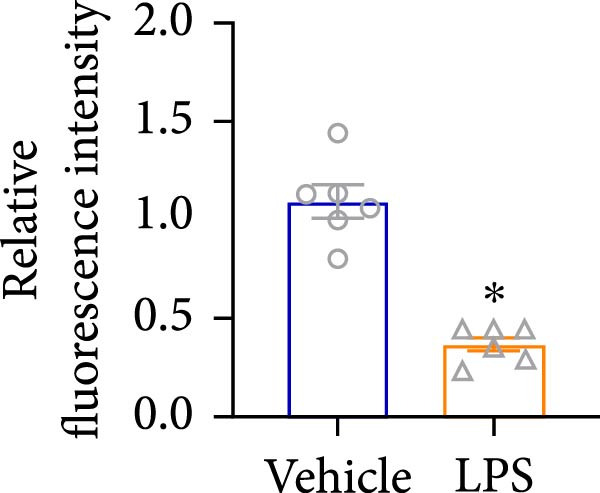
(F)

**Figure:**
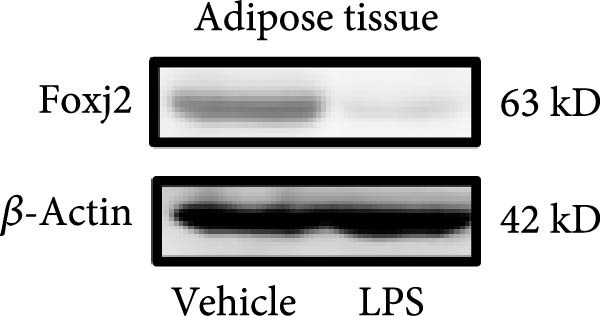
(G)

**Figure:**
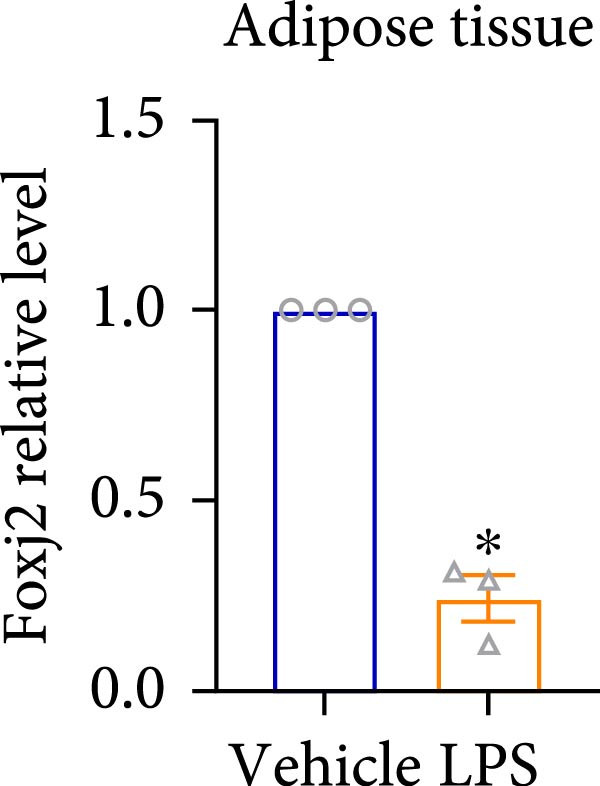
(H)

**Figure:**
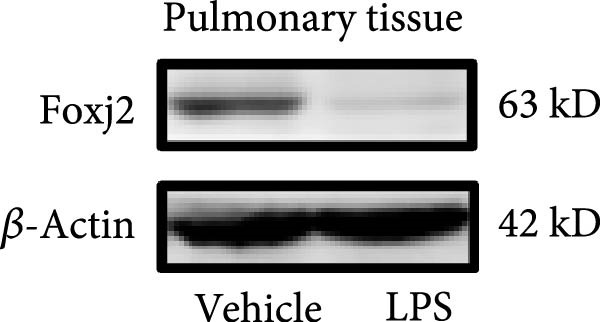
(I)

**Figure:**
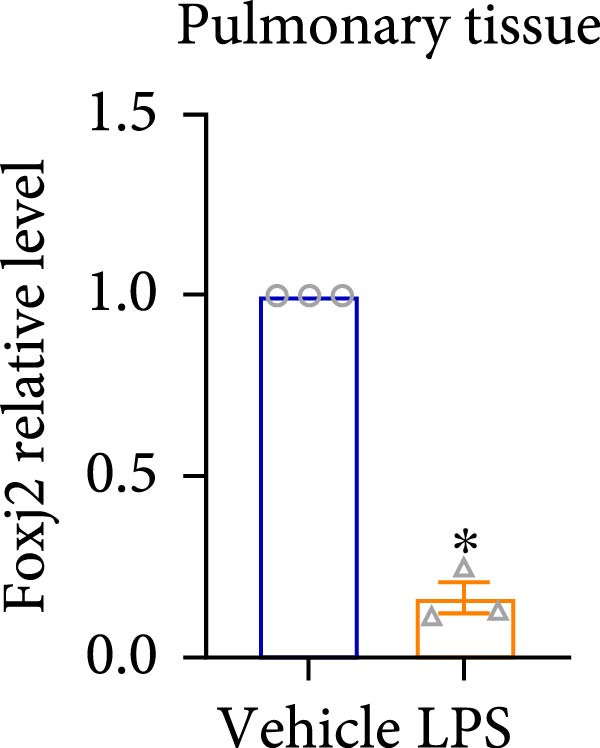
(J)

### 3.3. Foxj2 Regulates Macrophage Polarization

To decipher the functional implications of Foxj2 in regulating inflammatory and immune responses triggered by LPS in macrophages, Foxj2 expression levels were manipulated using adenovirus‐mediated gene overexpression. As shown in Supporting Information 2: Figure S2, Ad‐Foxj2 infection significantly increased Foxj2 expression in macrophages. Upon overexpression of Foxj2, no changes were observed in the basal production levels of key inflammatory mediators like TNF, IL‐1β, IL‐6, and IL‐12. However, a noticeable effect emerged when examining macrophages activated through LPS exposure. Ectopic expression of Foxj2 was found to suppress the excessive activation of all these inflammatory factors at both 4 and 20 h after LPS stimulation. Remarkably, at the 20 h time point, the levels of these cytokines were significantly reduced in the Foxj2‐overexpressing group compared with the control group (Figure 3A–D). Additionally, the induction of type I IFNs and IFN‐dependent genes by LPS was also influenced by the induced expression of Foxj2. Notably, the expression levels of INF‐stimulated gene 15 (ISG15) and IFN‐β, both known to be upregulated by type I IFNs, were attenuated in LPS‐activated macrophages with increased Foxj2 expression (Figure 3E,F). To evaluate the role of Foxj2 in the production of inflammatory cytokines induced by LPS, we investigated the effect of Foxj2 on the secretion of TNF, IL‐1β, IL‐6, IL‐12, ISG15, and IFN‐β. Detection via ELISA showed that after the addition of LPS, the cells began to produce TNF‐α and IL‐6. Overexpression of Foxj2 significantly inhibited the LPS‐induced secretion levels of TNF‐α and IL‐6 proteins (Figure 3G,L). Overall, the current data provided evidence supporting an antiinflammatory role for Foxj2 in macrophages, indicating its potential function in dampening the inflammatory response during LPS‐mediated activation.

Figure 3Foxj2 regulates macrophage polarization. (A–F) The influence of elevated Foxj2 levels on LPS‐induced inflammatory factor expression in cultured peritoneal macrophages was assessed using reverse‐transcription quantitative PCR. (G–L) The influence of elevated Foxj2 levels on LPS‐induced inflammatory factor secretion in cultured peritoneal macrophages was assessed using ELISA. Data are presented as the mean ± SEM (*n* = 3).  ^∗^ *p*  < 0.05 vs. Ad‐EV group. Foxj2, forkhead box J2; Ad‐EV, adenovirus‐empty vector.

**Figure:**
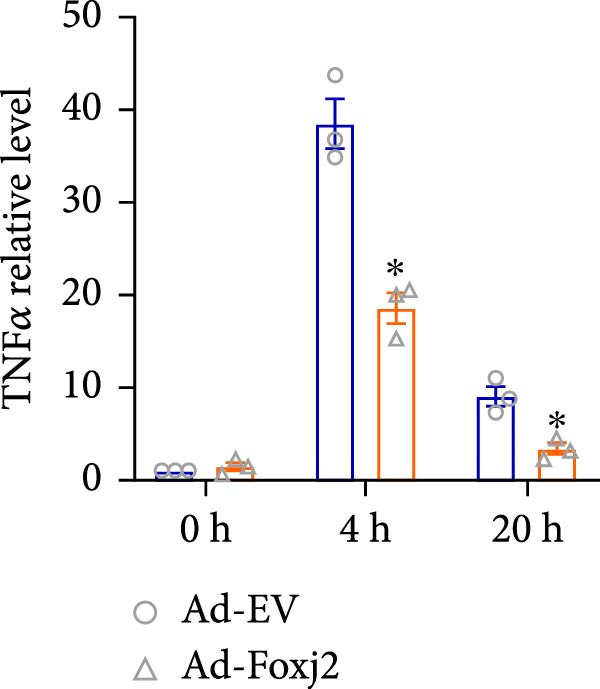
(A)

**Figure:**
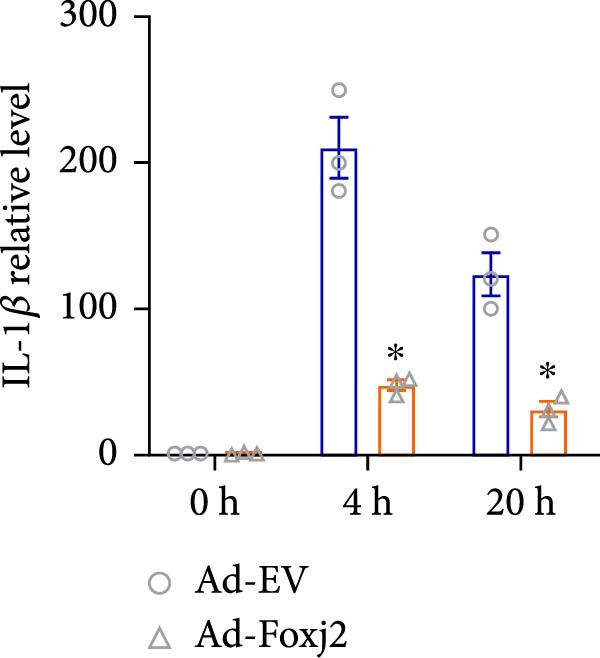
(B)

**Figure:**
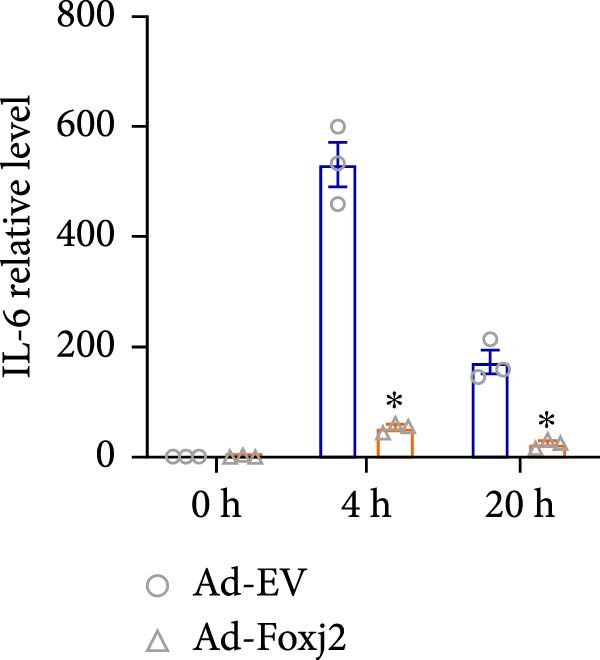
(C)

**Figure:**
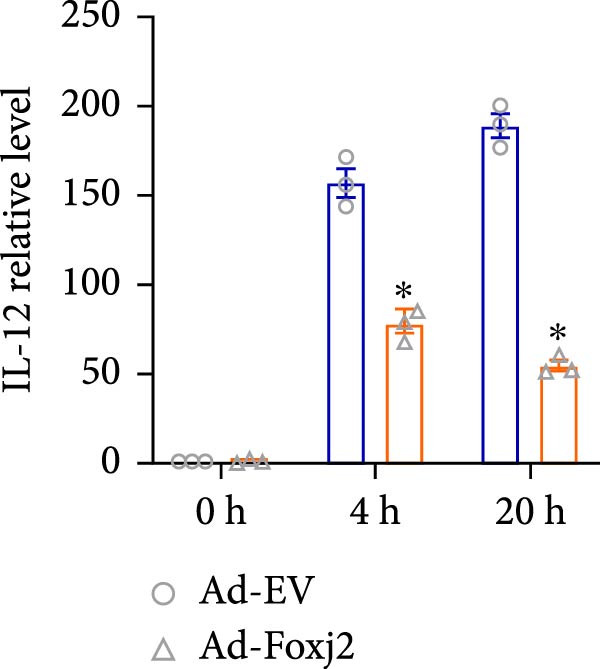
(D)

**Figure:**
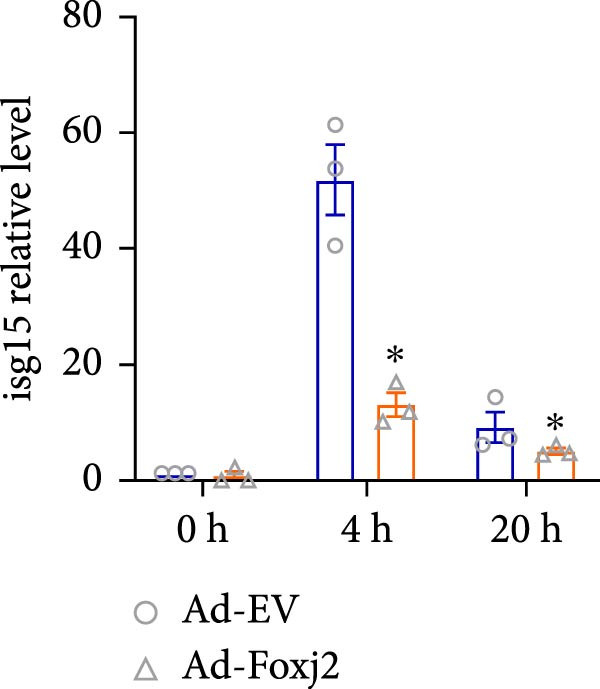
(E)

**Figure:**
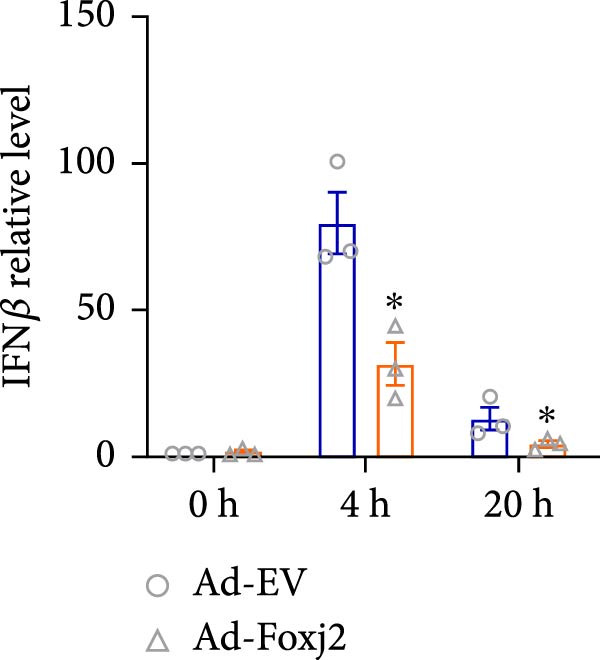
(F)

**Figure:**
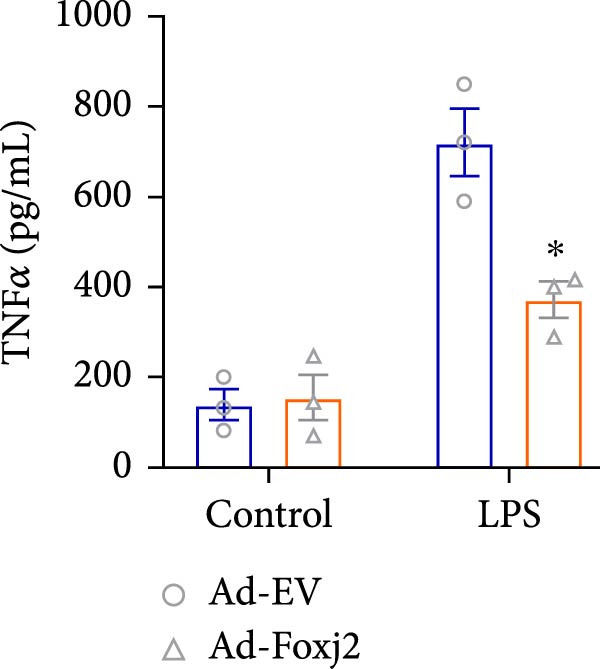
(G)

**Figure:**
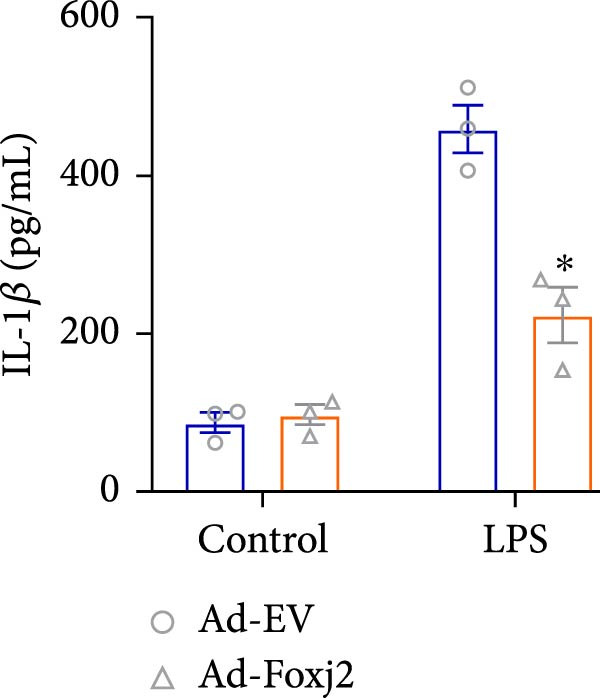
(H)

**Figure:**
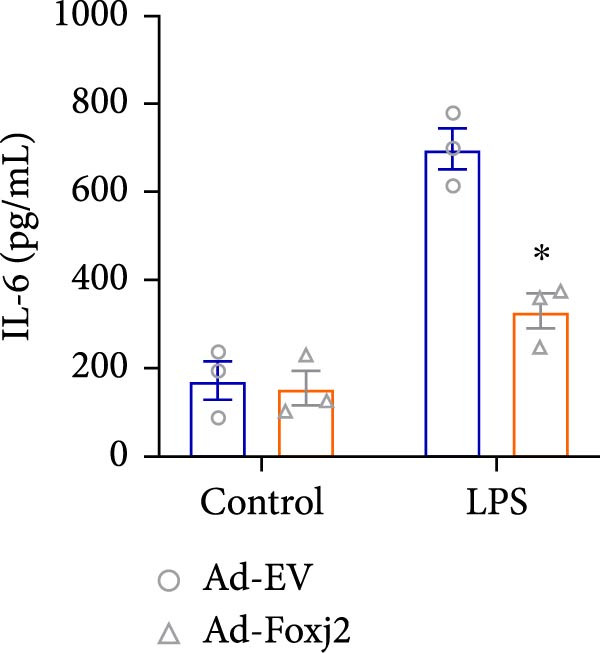
(I)

**Figure:**
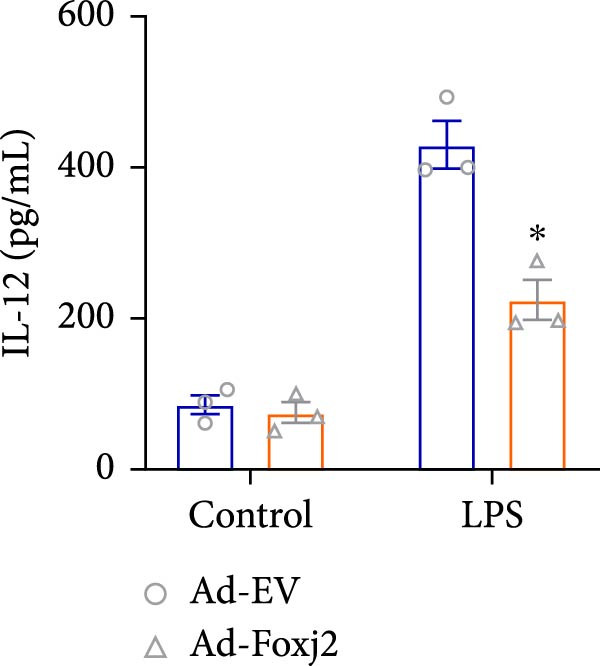
(J)

**Figure:**
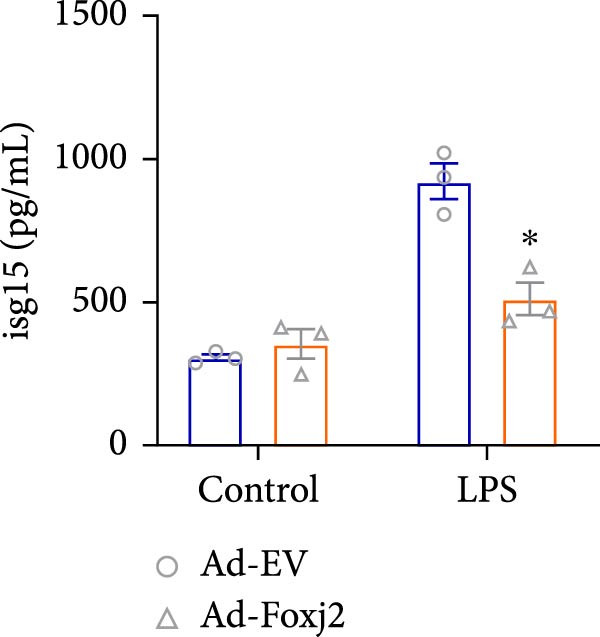
(K)

**Figure:**
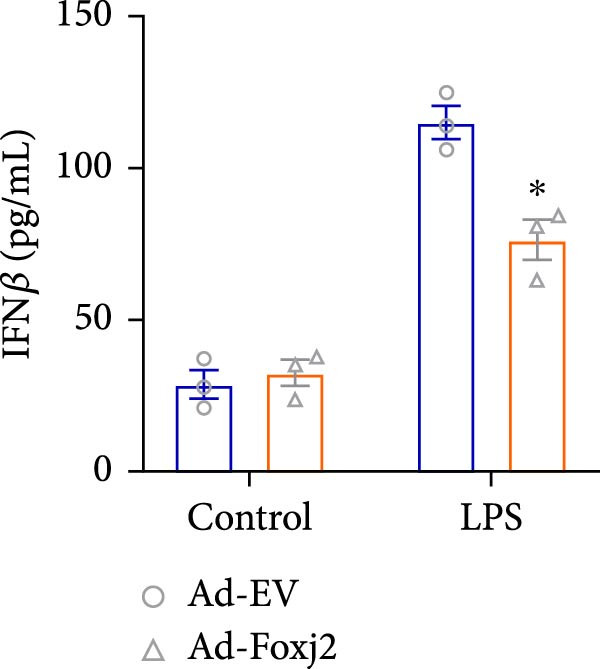
(L)

Unlike LPS, IL4, which promotes the differentiation of alternatively activated macrophages, increased the Foxj2 level, as shown in Supporting Information 1: Figure S1A,B. The role of Foxj2 in the mRNA expression of classical M2 markers and cytokines induced by IL4 was further investigated. In macrophages that ectopically expressed Foxj2, IL4 stimulation resulted in a significantly enhanced and faster increase in the mRNA levels of Arginase 1, CD206, CD30, and CCL24, while Foxj2 overexpression amplified the effect of IL4 on macrophages (Supporting Information 1: Figure S1D–H).

### 3.4. Effects of Foxj2 on Inflammatory Signaling Pathways

Given the well‐established roles of Stat1, NF‐κB and MAPK signaling pathways in mediating inflammatory responses in LPS‐stimulated macrophages, the present study sought to establish any potential interplay between Foxj2 and the phosphorylation levels of key signaling proteins within these pathways. As evidenced in Figure 4A, upon LPS stimulation, the phosphorylation levels of Jnk, Erk1/2 and p38 kinases within the MAPK pathway peaked at 0.5 h post‐treatment, before declining sharply back to baseline levels after 6 h. Meanwhile, the phosphorylation of p65, a central component of the NF‐κB pathway, reached its maximum at 2 h after LPS induction and returned to baseline after 10 h. The phosphorylation of Stat1, a key player in the Stat1 pathway, peaked at 6 h post‐LPS administration. Interestingly, upon exogenous overexpression of Foxj2, a significant reduction in the phosphorylation levels of Stat1, p65, Erk1/2, Jnk, and p38 was observed. These findings collectively suggested that Foxj2 functions as a negative regulator, dampening the activation of the Stat1, NF‐κB, and MAPK signaling cascades, thereby contributing to the inhibition of inflammatory gene upregulation in LPS‐activated macrophages. To explore whether Foxj2 could also directly regulate transcription of Tak1, we established the promoter‐luciferase reporter constructs containing the sequences ranging from upstream 2000 base pairs (bp) to downstream 200 bp of gene transcription initiation sites. The dual luciferase reporter gene assay was conducted in HEK293 cells. As depicted in Figure 4B, Foxj2 overexpression significantly reduced the promoter activity of Tak1. To determine whether Foxj2 showed high enrichment in promoters of Tak1, ChIP was performed in Raw264.7. By bioinformatics analysis, several putative Foxj2 binding sites, which contained the consensus sequence of 5′‐ATAAATAA‐3′ or 5′‐ATAAACAT‐3′, were found in Tak1 promoter ranging from −2000 to +200 bp, and they are denoted as “seq‐No.” in Figure 4C. As expected, no significant occupancy of Foxj2 on the promoters was observed in cells infected with Ad‐EV. However, for the Ad‐Foxj2 group, Foxj2 enrichment in the specific binding sites of Tak1 gene promoter was observed (Figure 4D).

Figure 4Foxj2 inhibits LPS‐induced inflammatory responses in macrophages. (A) The effect of Foxj2 overexpression on the activation of Stat1, NF‐κB and MAPK pathways through LPS induction in peritoneal macrophages was revealed through Western blotting. (B) The luciferase reporter gene vector containing the Tak1 promoter was co‐transfected into HEK293 cells together with the internal control plasmid pRL‐TK. Subsequently, the cells were infected with Ad‐Foxj2 or Ad‐EV, and the relative luciferase activity was detected. (C)Bioinformatics analysis was conducted to unveil the DNA‐binding motifs of Foxj2. (D) ChIP assay was performed in Raw264.7 cells. IgG was used as a negative control. The putative Foxj2 binding sites of each Tak1 promoter were denoted as “seq‐No.”. Values are expressed as percent of amplified signals from IP chromatin to amplified input signals obtained from the same sample. Data are presented as the mean ± SEM standard deviation of the mean (*n* = 3).  ^∗^ *p*  < 0.05 vs. Anti‐IgG group, *p*  < 0.05 vs. Ad‐EV group. Foxj2, forkhead box J2; LPS, lipopolysaccharide; Ad‐EV, adenovirus‐empty vector.

**Figure:**
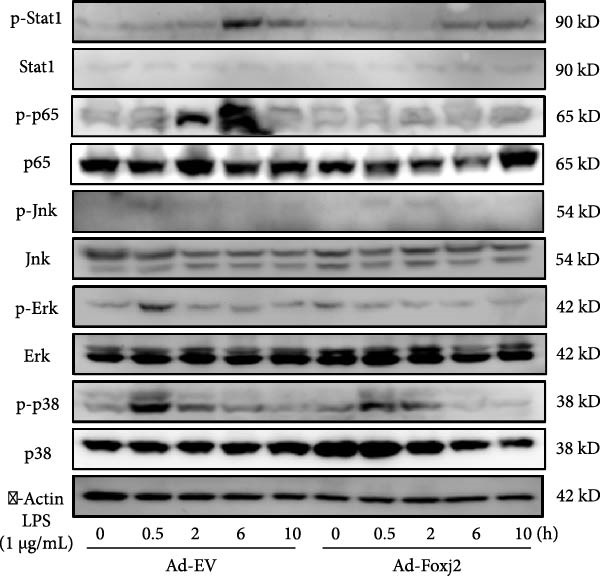
(A)

**Figure:**
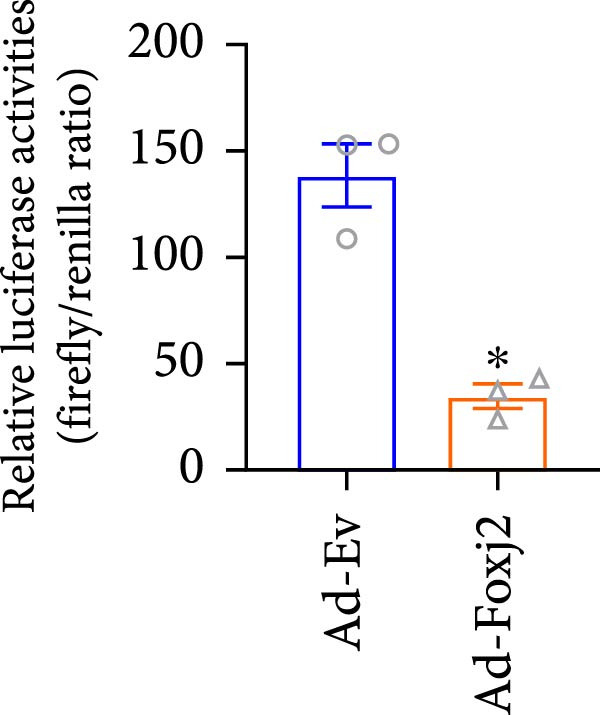
(B)

**Figure:**
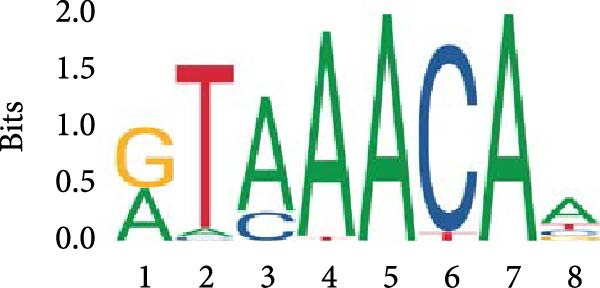
(C)

**Figure:**
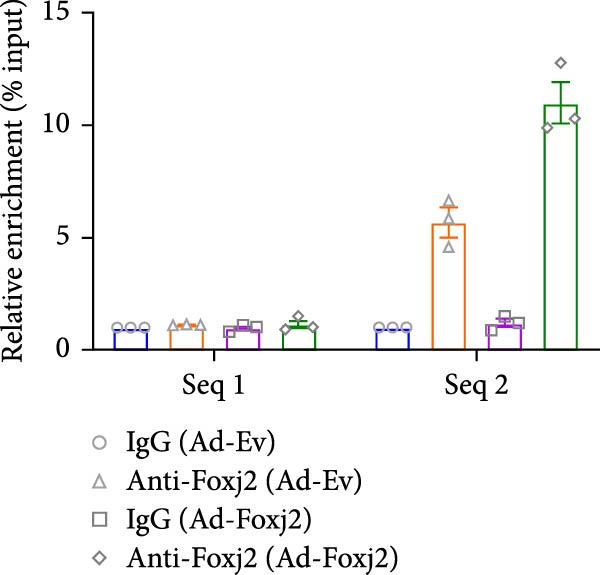
(D)

### 3.5. Involvement of Foxj2 in Other Inflammatory Diseases

Considering the crucial regulatory role of Foxj2 in inflammatory processes within mice experiencing endotoxemia, the present authors further explored its potential involvement in other inflammatory diseases. Unexpectedly, it was observed a decline in Foxj2 mRNA expression levels in the adipose tissue of mice with obesity induced by a high‐fat diet (HFD) (Figure 5A), as well as in the livers of mice with nonalcoholic fatty liver disease (NAFLD) induced by HFD (Figure 5B). However, contrasting this observation, no significant change in Foxj2 mRNA expression was measured in the aorta and kidneys of streptozotocin (STZ)‐induced diabetic mice compared with the control group (Figure 5C,D). In other disease models, differential mRNA expressions of Foxj2 were noticed. In the context of STZ‐induced diabetes, doxorubicin (Dox)–induced cardiomyopathy, acute myocardial infarction (AMI), and D‐galactose (D‐gal) induced aging conditions, differences in Foxj2 mRNA levels were indeed observed between the myocardium of healthy control mice and those affected by the respective diseases (Figure 5E–H). The current results suggested that Foxj2 may play diverse and potentially context‐specific roles in various inflammatory diseases.

Figure 5Involvement of Foxj2 in other inflammatory diseases. (A–B) C57BL/6J mice (male; 6‐week‐old) were allocated for 12 weeks to either a standard chow diet or a high‐fat diet with 60% of its calories from fat. The mice were euthanized and Foxj2 mRNA levels in (A) adipose tissue and (B) liver were detected. (C–E) C57BL/6J mice (male; 6‐week‐old) received an intraperitoneal injection of 50 mg/kg/day STZ for 5 consecutive days. Control mice, matched in age, were administered an equivalent volume of the vehicle solution. 1 week following the STZ injection, mice were euthanized at 8 weeks of age, Foxj2 mRNA expression in (C) aorta, (D) kidney and (E) myocardium was assessed. (F) C57BL/6J mice (male; 6‐week‐old) received intraperitoneal injections of Dox at a dosage of 2.5 mg/kg per injection on six occasions over 2 weeks. Four months after the final Dox injection, the mice were euthanized and Foxj2 mRNA expression in the myocardium was examined. (G) C57BL/6J mice (male; 8‐week‐old) underwent left‐anterior descending coronary artery ligation to induce myocardial infarction, with a sham‐operated group serving as the control. At 24 h after surgery, the mice were euthanized and Foxj2 mRNA expression in the myocardium was assessed. (H) C57BL/6J mice (male; 8‐week‐old) were injected with D‐gal (120 mg/kg/d) for 12 weeks continuously. Foxj2 mRNA expression in the myocardium was detected in D‐gal‐induced aging mice and control group mice. Data are presented as the mean ± SEM (*n* = 6).  ^∗^ *p*  < 0.05. Foxj2, forkhead box J2; STX, streptozotocin; Dox, doxorubicin; D‐gal, D‐galactose.

**Figure:**
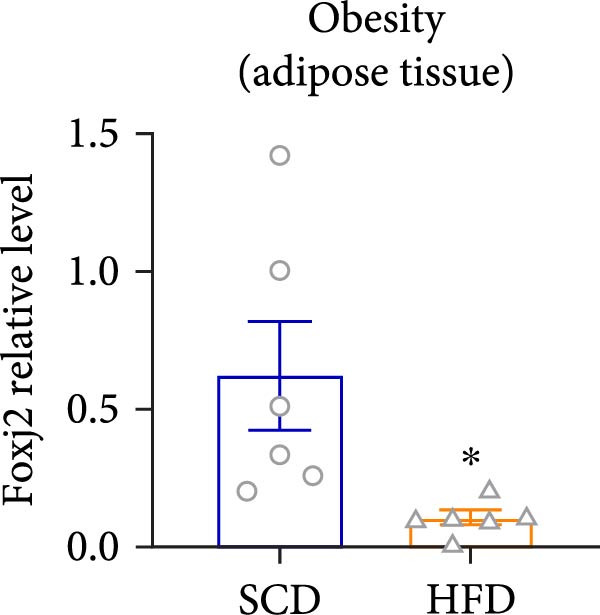
(A)

**Figure:**
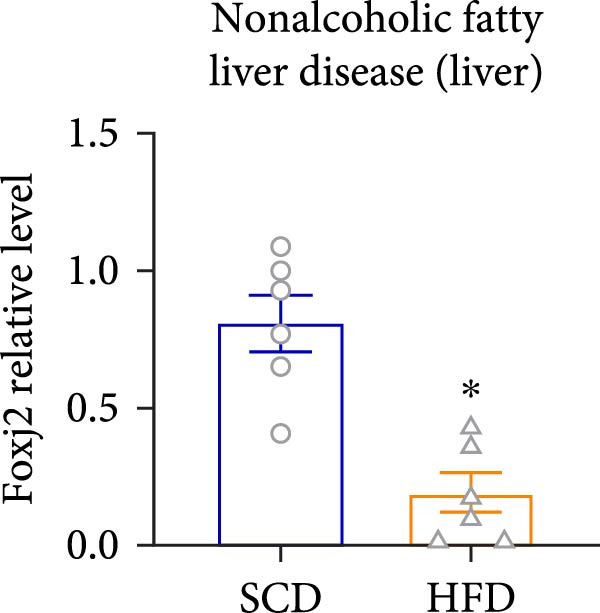
(B)

**Figure:**
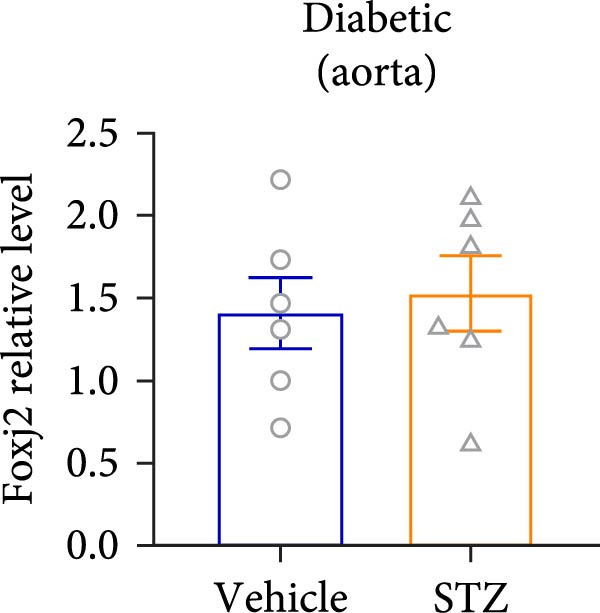
(C)

**Figure:**
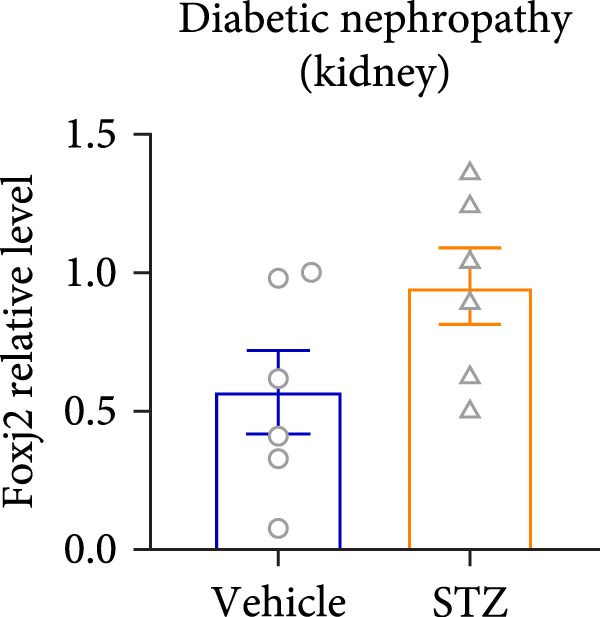
(D)

**Figure:**
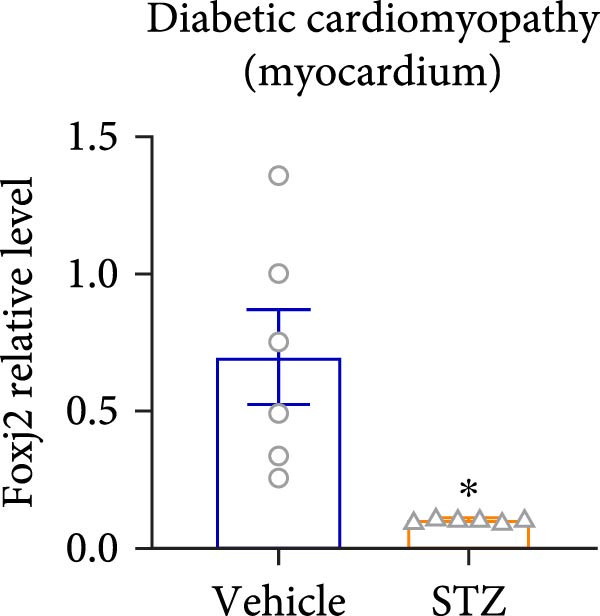
(E)

**Figure:**
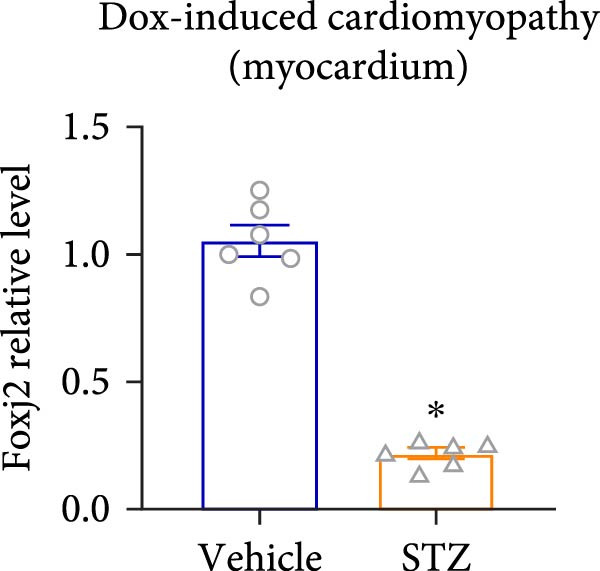
(F)

**Figure:**
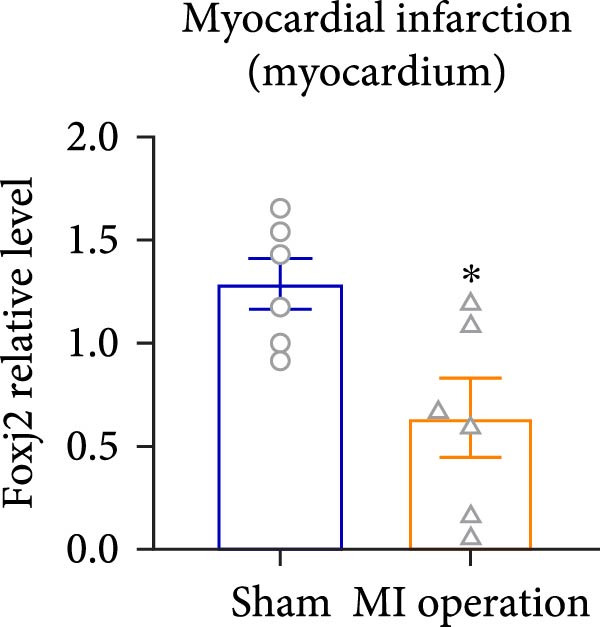
(G)

**Figure:**
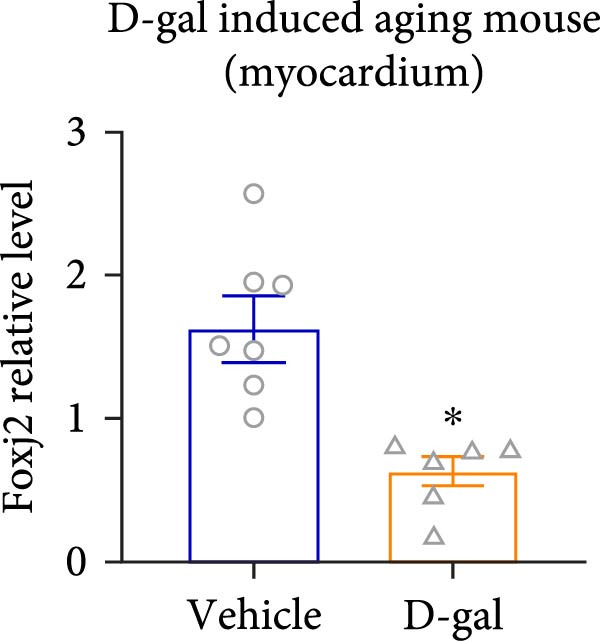
(H)

## 4. Discussion

Extensive research consistently pointed towards the involvement of the forkhead box/hepatocyte nuclear factor 3 family of transcription factors in modulating inflammatory processes [17]. Therefore, the present study focused on the role of Foxj2, a member of this family, in the context of LPS stimulation in macrophages. The current findings revealed that LPS treatment led to a significant decrease in Foxj2 expression levels. It was further demonstrated that Foxj2 plays a critical anti‐inflammatory role since its restoration could mitigate the inflammatory responses induced by LPS. This attenuation was achieved by suppressing key signaling pathways such as Stat1, NF‐κB, and MAPK. Therefore, the present work highlights Foxj2 as a potent negative regulator of inflammation in macrophages responding to LPS induction.

Macrophage activation encompasses a spectrum of states, with the classical M1 phenotype induced by LPS and the alternative M2 phenotype induced by IL4, representing the two extremes. The polarization of macrophages between M1 and M2 states is a regulated process involving a network of signaling pathways, transcriptional controls, and post‐transcriptional mechanisms [26]. M1 macrophages, known for their pro‐inflammatory functions, play a crucial role in defending the host against pathogens, while M2 macrophages contribute to tissue repair and maintaining homeostasis [27]. In the present study, Foxj2 expression was significantly decreased at 2 h and peaked at 6 h after LPS stimulation. Upon Foxj2 was overexpressed, inflammatory pathways remained activated after 6 and 10 h, and there was a notable surge in inflammatory factors after 20 h, contrasting with the control group where levels plummeted rapidly, revealing that upregulating Foxj2 significantly decreased the M1 macrophage activation in vivo. Ectopic expression of Foxj2 was found to suppress the excessive activation of inflammatory factors at both 4 and 20 h post‐LPS stimulation. Remarkably, at the 20 h time point, the levels of these cytokines were significantly reduced in the Foxj2‐overexpressing group compared with the control group, suggesting that the presence of Foxj2 curtailed systemic inflammation in septic mice and counteracted the inflammatory response triggered by LPS in macrophages. The present results indicate that the initiation of inflammation in endotoxin‐activated macrophages may be attributed at least in part to a decrease in Foxj2 levels. The activation of inflammatory cytokines in macrophages stimulated by LPS preceded that of Foxj2, which might be attributed to the swift initiation of the inflammatory cascade preceding the reduction of Foxj2 levels upon LPS stimulation.

To investigate the potential conserved role of Foxj2 in macrophages across different tissues, we examined its expression patterns in both adipose and lung macrophages under inflammatory conditions. Immunofluorescence staining revealed a significant downregulation of Foxj2 in CD68‐positive macrophages isolated from adipose tissue and lung tissue of LPS‐challenged mice. This regulatory pattern aligns with our observations in peritoneal macrophages, collectively suggesting that Foxj2 may represent a common regulatory node in macrophage responses to inflammatory stimuli. The consistent downregulation of Foxj2 across these distinct macrophage populations provides compelling evidence for its conserved regulatory role in the innate immune response. It should be noted that this study has certain limitations. Functional validation experiments were primarily conducted in peritoneal macrophages, with parallel studies yet to be performed in macrophages from different tissue origins (such as alveolar macrophages or Kupffer cells). Furthermore, no distinction was made between different subpopulations, including tissue‐resident macrophages and monocyte‐derived macrophages. Future investigations exploring Foxj2 function in other tissue‐resident macrophages, would be valuable to further elucidate its broad significance in inflammatory regulation. Tak1 is a member of the MAPKKK family and is activated by various cytokines, including transforming growth factor TGF‐β and IL‐1 [28]. Tak1 has been reported to play a role in LPS‐induced NF‐κB activation [29]. Some previous studies also indicated that Tak1 plays a central and essential role in LPS‐induced activation of p38 and JNKin pre‐B and myeloid lineage cell lines [28, 30] Several recent reports said that Tak1 could also activate the MEK1/2‐ERK1/2 pathways [31, 32]. Thus, Therefore, we focused on the effect of Foxj2 on Tak1 during the inflammatory response. We found that Foxj2 directly binds to the promoter region of the Tak1 gene, and overexpression of Foxj2 can effectively suppress the promoter activity of Tak1. Although our data indicate that Foxj2 directly suppresses Tak1 transcription, we cannot completely rule out the possibility that Foxj2 may additionally influence Tak1 expression through post‐transcriptional regulatory mechanisms. Foxj2 likely alleviates inflammation primarily by repressing Tak1 transcription, thereby inhibiting the NF‐κB and MAPK pathways. As for the STAT1 signaling pathway, Foxj2 may exert its regulatory effects through both Tak1‐dependent and Tak1‐independent mechanisms. This function of Foxj2 may represent a crucial “brake” mechanism in vivo, which prevents excessive activation of the immune system when responding to infections, thereby avoiding damage to host tissues. In sepsis, impaired function of Foxj2 may lead to uncontrolled Tak1 signaling, which in turn drives “cytokine storms” and chronic inflammation. Further validation using Tak1 knockdown experiments could be pursued in future studies, though such investigations fall beyond the primary focus of the present work on core mechanisms.

It is noteworthy that the role of Foxj2 in regulating inflammation is not confined to just myeloid cells. The reduction of Foxj2 levels induced by LPS was observed in a variety of mouse tissues such as the heart, liver, spleen, lung, kidney, adipose tissue, and blood vessels. Our findings indicate that Foxj2 is involved in the regulation of inflammatory responses and suggest its potential as a candidate for further investigation under septic conditions. Moreover, in the in vivo animal experiment, a decrease in Foxj2 levels was observed in the pathological process of several inflammation‐related diseases, including HFD‐induced obesity, NAFLD, Dox‐induced cardiomyopathy, AMI and D‐gal induced aging conditions, suggesting that Foxj2 may play diverse and potentially context‐specific roles in various inflammatory diseases. For a more detailed exploration of the role of Foxj2 in specific cell types within certain tissues during endotoxemia, future studies utilizing macrophage‐specific conditional knockout models will be essential to validate the therapeutic potential of targeting Foxj2 in vivo.

## 5. Conclusions

In summary, the current study substantiated that Foxj2 is involved in the regulation of inflammatory responses. Through molecular mechanism investigations, it was demonstrated that the enhanced Foxj2 expression may exert its influence on macrophage polarization and inflammation by modulating the activity of Stat1, NF‐κB, and MAPK signaling pathways. These findings contribute novel insights into the potential role of Foxj2 as a promising therapeutic target for inflammatory processes that drive the progression of this life‐threatening condition. Foxj2 represents a promising candidate target worthy of further investigation under septic conditions, as manipulating its expression levels may alleviate the severe inflammatory responses characteristic of such diseases, thereby potentially improving patient outcomes.

## Ethics Statement

The animal experiments were conducted with the approval of the Ethics Committee of Tongji Medical College, Huazhong University of Science and Technology (IACUC Number: 4012), and adhered to NIH guidelines for animal care and use.

## Conflicts of Interest

The authors declare no conflicts of interest.

## Author Contributions

Pianpian Huang was responsible for the design and execution of the study, data analysis, and the original manuscript draft. Jun Fu and Ji Hu participated in the experimental work and contributed to the manuscript preparation. Jun Fu and Yinghong Lei was involved in both manuscript writing and data analysis. Caobo Dai and Tingyu Wu provided substantial contributions to data analysis and interpretation. Ju Liu was the principal investigator who conceived the study, participated in its design, and assisted in drafting the manuscript. Pianpian Huang, Jun Fu, and Ji Hu contributed equally to this work.

## Funding

The work was supported by the Natural Science Foundation of Hubei Province (Grant 2023AFB533), Wuhan Natural Science Foundation Exploration Plan Key Clinical Research Projects in Municipal Medical Institutions (Grant 2024020801020392).

## Supporting Information

Additional supporting information can be found online in the Supporting Information section.

## Supporting information


**Supporting Information 1** Figure S1. Foxj2 expression is induced by IL4 and thereby promotes IL4‐induced macrophage M2 polarization. (A) Protein expression levels of Foxj2 in peritoneal macrophages exposed to various concentrations of IL4 for 48 h. (B) Temporal expression pattern of Foxj2 protein in peritoneal macrophages cultured with IL4 (20 ng/mL). (C–H) Effects of Foxj2 overexpression on the mRNA expression levels of M2 markers and cytokines induced by IL4 in cultured peritoneal macrophages, as determined through reverse‐transcription quantitative PCR. Data are presented as the mean ± SEM (*n* = 4).  ^∗^ *p* < 0.05 vs. Ad‐EV group. Foxj2, forkhead box J2. Ad‐EV, adenovirus‐empty vector.

**Supporting Information 2** Figure S2. Adenovirus vector infection significantly increases Foxj2 expression in macrophages. (A) The expression level of Foxj2 mRNA in macrophages of Ad‐Ev and Ad‐Foxj2. (B) Protein expression level of Foxj2 in macrophages of Ad‐Ev and Ad‐Foxj2. Data are presented as the mean ± SEM (*n* = 3).  ^∗^ *p* < 0.05 vs. Ad‐EV group. Foxj2, forkhead box J2. Ad‐EV, adenovirus‐empty vector.

## Data Availability

The data utilized in this study can be accessed by contacting the corresponding author upon reasonable request.
