# Supplementary figures and images for: Foxj2 Attenuates LPS‐Induced Inflammatory Response in Macrophages
Source: Mediators Inflamm. 2025 Dec 11;2025:3854538. doi: 10.1155/mi/3854538 (PMC12767408; doi:10.1155/mi/3854538)

## Slide 1
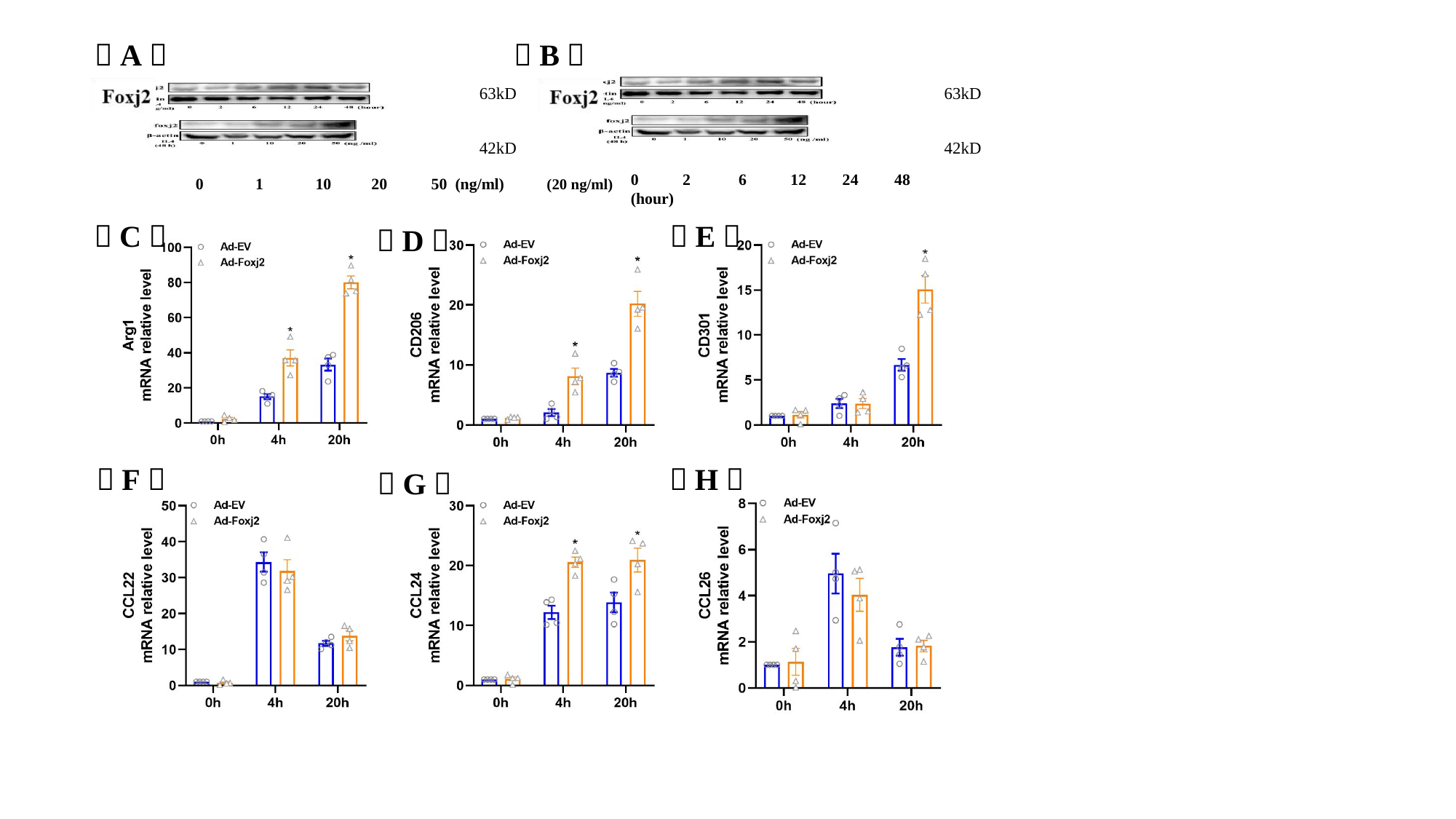

（A）
（B）
63kD
63kD
42kD
42kD
0 2 6 12 24 48 (hour)
0 1 10 20 50 (ng/ml)
(20 ng/ml)
（C）
（E）
（D）
（F）
（H）
（G）

Supplement: Supplementary file 1 — Supporting Information 1 Figure S1. Foxj2 expression is induced by IL4 and thereby promotes IL4‐induced macrophage M2 polarization. (A) Protein expression levels of Foxj2 in peritoneal macrophages exposed to various concentrations of IL4 for 48 h. (B) Temporal expression pattern of Foxj2 protein in peritoneal macrophages cultured with IL4 (20 ng/mL). (C–H) Effects of Foxj2 overexpression on the mRNA expression levels of M2 markers and cytokines induced by IL4 in cultured peritoneal macrophages, as determined through reverse‐transcription quantitative PCR. Data are presented as the mean ± SEM (n = 4). ∗ p < 0.05 vs. Ad‐EV group. Foxj2, forkhead box J2. Ad‐EV, adenovirus‐empty vector. [file MI-2025-3854538-s002.pptx]

## Slide 1
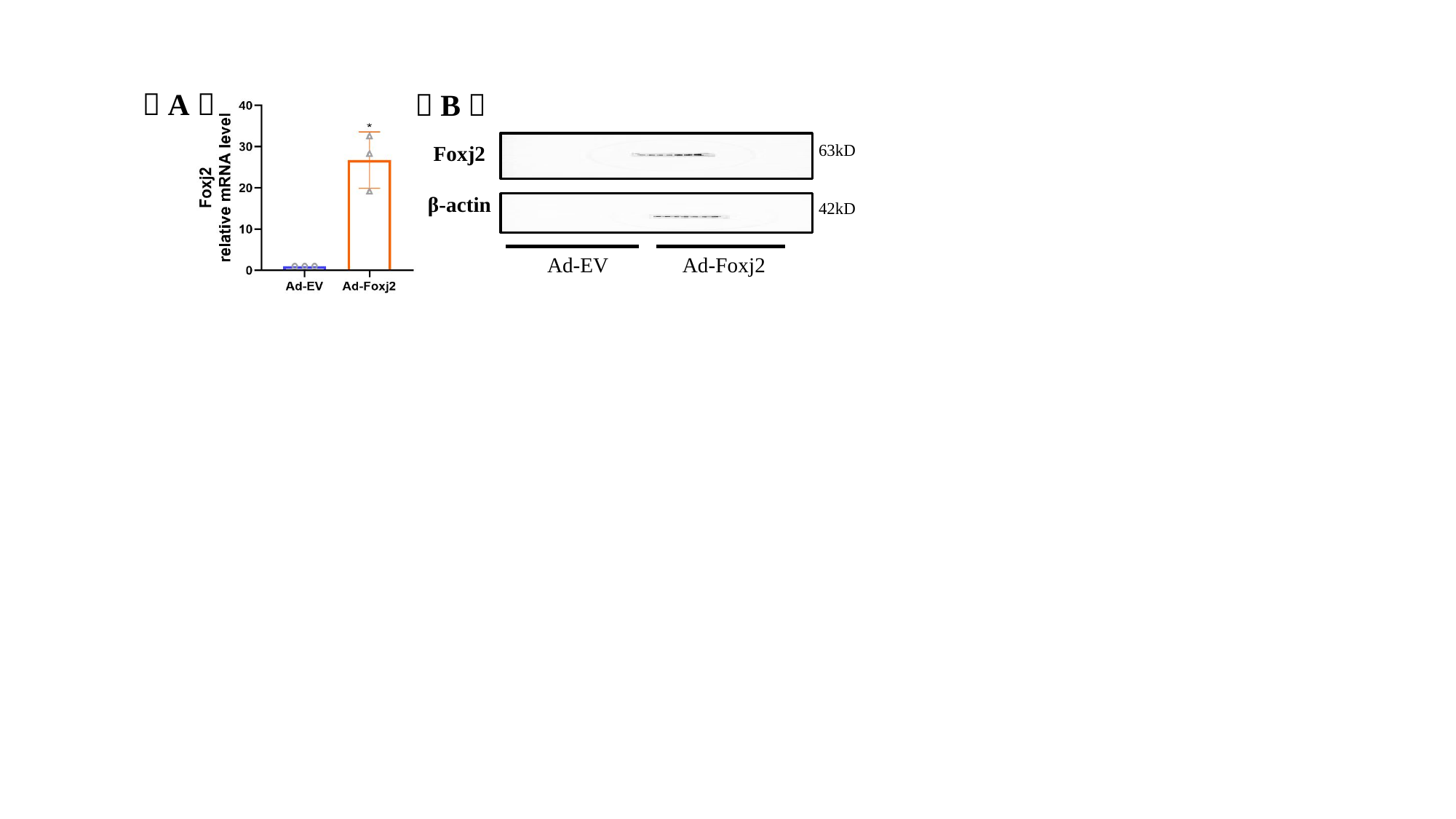

（A）
（B）
Foxj2
63kD
β-actin
42kD
Ad-EV
Ad-Foxj2

Supplement: Supplementary file 2 — Supporting Information 2 Figure S2. Adenovirus vector infection significantly increases Foxj2 expression in macrophages. (A) The expression level of Foxj2 mRNA in macrophages of Ad‐Ev and Ad‐Foxj2. (B) Protein expression level of Foxj2 in macrophages of Ad‐Ev and Ad‐Foxj2. Data are presented as the mean ± SEM (n = 3). ∗ p < 0.05 vs. Ad‐EV group. Foxj2, forkhead box J2. Ad‐EV, adenovirus‐empty vector. [file MI-2025-3854538-s001.pptx]
